# CIRCADIAN CLOCK-ASSOCIATED 1 represses thermotolerance by inhibiting *HEAT SHOCK FACTOR A2* expression in nonheading Chinese cabbage

**DOI:** 10.1093/hr/uhag033

**Published:** 2026-02-09

**Authors:** Ying He, Dong Xiao, Xlin Hou, Yiran Li, Hongfang Zhu

**Affiliations:** National Key Laboratory of Crop Genetics & Germplasm Innovation and Utilization, Key Laboratory of Biology and Genetic Improvement of Horticultural Crops (East China), Ministry of Agriculture and Rural Affairs of China, Engineering Research Center of Germplasm Enhancement and Utilization of Horticultural Crops, Ministry of Education of China, Nanjing Agricultural University, Nanjing 210095, China; Engineering and Technical Center for Modern Horticulture, Jiangsu Vocational College of Agriculture and Forestry, Jurong 212400, China; National Key Laboratory of Crop Genetics & Germplasm Innovation and Utilization, Key Laboratory of Biology and Genetic Improvement of Horticultural Crops (East China), Ministry of Agriculture and Rural Affairs of China, Engineering Research Center of Germplasm Enhancement and Utilization of Horticultural Crops, Ministry of Education of China, Nanjing Agricultural University, Nanjing 210095, China; The Sanya Institute of Nanjing Agricultural University, Nanjing Agricultural University, Sanya 572000, China; National Key Laboratory of Crop Genetics & Germplasm Innovation and Utilization, Key Laboratory of Biology and Genetic Improvement of Horticultural Crops (East China), Ministry of Agriculture and Rural Affairs of China, Engineering Research Center of Germplasm Enhancement and Utilization of Horticultural Crops, Ministry of Education of China, Nanjing Agricultural University, Nanjing 210095, China; National Key Laboratory of Crop Genetics & Germplasm Innovation and Utilization, Key Laboratory of Biology and Genetic Improvement of Horticultural Crops (East China), Ministry of Agriculture and Rural Affairs of China, Engineering Research Center of Germplasm Enhancement and Utilization of Horticultural Crops, Ministry of Education of China, Nanjing Agricultural University, Nanjing 210095, China; Shanghai Key Laboratory of Protected Horticultural Technology, Horticulture Research Institute, Shanghai Academy of Agricultural Sciences, Shanghai 201403, China

## Abstract

In the context of global warming, elevated temperatures present serious challenges to the growth, quality, and productivity of nonheading Chinese cabbage (NHCC). Understanding the mechanisms underlying thermotolerance in NHCCs is therefore critically important. In this study, we investigated the influence of heat stress (HS) duration and circadian rhythm on gene expression using time-resolved transcriptome sequencing. The results showed that during the early stages of HS, NHCC primarily engaged in physiological processes such as stimulus perception and signal transduction. In contrast, prolonged HS exposure activated antioxidant metabolism, reduced photosynthetic capacity, and accelerated leaf senescence. Weighted gene coexpression network analysis (WGCNA) further revealed a strong link between circadian regulation and HS responses. Notably, our findings demonstrate that the core circadian clock component CIRCADIAN CLOCK ASSOCIATED 1 (BcCCA1) negatively regulated heat tolerance by repressing the transcription of *BcHSFA2*. Collectively, these results provide new insights into the molecular mechanisms underlying HS responses in NHCCs and highlight the regulatory role of circadian rhythms in plant thermotolerance.

## Introduction

Nonheading Chinese cabbage [*Brassica campestris* (syn*. Brassica rapa*) ssp*. chinensis*, Pak-choi] (NHCC) is one of three subspecies within the *Brassica* genus of the *Cruciferae* family [1]*.* Originating from China, it boasts a long cultivation history and thrives at temperatures ranging from 18°C to 22°C. Owing to its short growth cycle, high yield, and rich nutritional content, it has been widely cultivated across Southeast Asia, Europe, and America, making it a mainstream vegetable worldwide. However, it tends to decrease in productivity when exposed to elevated temperatures [2]. Thus, clarifying the molecular processes driving heat stress (HS) responses is essential for increasing the agronomic effectiveness of NHCC [3].

Both internal and external factors influence plant resistance to HS. External factors include temperature intensity, duration, and rate of change [4, 5]. The internal factors affecting plant tolerance to HS include genetic type, chronological age, growth phase, tissue category, circadian phase, and previous contact with heat or other stressors [6–10]. In particular, prolonged heat exposure and brief heat shock differentially impact plant physiology, metabolism, and gene expression [11, 12]. HS represents a growing environmental pressure that affects plant physiology and molecular processes, such as photosynthesis [13], oxidative damage [14], DNA methylation [15], histone modification [16], chromatin remodeling [15], alternative splicing [17], translation [18], and protein degradation [19]. Therefore, understanding the transcriptome dynamics of plants exposed to different durations of HS is highly important for revealing how to cope with different durations of HS [20]. Xu *et al*. reported that NIGHT LIGHT-INDUCIBLE AND CLOCK-REGULATED 1 (LNK1) and NIGHT LIGHT-INDUCIBLE AND CLOCK-REGULATED 2 (LNK2) form complexes with the circadian clock components REVEILLE 4 (RVE4) and (REVEILLE 8) RVE8 to activate heat-responsive gene expression under high-temperature conditions. The morning-phased expression of *LNK*s and *RVE*s suggests that plants may exhibit enhanced responsiveness to high temperatures in the early morning [21]. Other scholars have proposed that plant responses to high temperatures are tightly regulated by the circadian rhythm. Specifically, high temperatures during the day (especially in the morning) are more likely to activate the heat response pathway, whereas during the evening and nighttime, clock proteins such as TIMING OF CAB EXPRESSION 1 (TOC1) and PSEUDO-RESPONSE REGULATOR 5 (PRR5) inhibit the heat response. This time-specific regulation not only affects thermomorphogenesis but is also directly associated with plant heat tolerance and viability [22–24]. On the basis of previous research findings, we infer that interactions occur between the HS response of plants and their circadian rhythm.

The plant circadian clock consists of three components: the input pathway, the core oscillator and the output pathway [25]. The output pathway regulates a diverse array of molecular and physiological processes, including flowering induction, photosynthesis, glucose metabolism, nutrient balance, auxin signaling, and responses to biotic and abiotic stresses such as HS and cold stress [26–28]. The regulatory network governing plant responses to high temperature substantially overlaps with the circadian rhythm [29]. Previous studies have shown that multiple circadian clock genes, such as *CIRCADIAN CLOCK-ASSOCIATED 1* (*CCA1*), *LONG ELONGATED HYPOCOTYL* (*LHY*), *GIGANTEA* (*GI*), *ZEITLUPE* (*ZTL*), and *PSEUDO-RESPONSE REGULATOR 7* (*PRR7*), are involved in temperature compensation [30–35]. Recently, numerous studies have investigated how different Brassica species withstand HS, approaching both physiological and molecular issues. For instance, Yu and colleagues examined physiological traits and how they are related to each other across twenty different varieties of rapeseed [36]. Yue and colleagues conducted a comprehensive study examining the gene coexpression networks within two distinct Chinese cabbage transcriptomes that demonstrated varying levels of heat resistance. Their research revealed critical metabolic pathways, pivotal functional modules, and central hub genes associated with prolonged HS adaptation [2]. Tabusam *et al*. reported that the expression of *HEAT SHOCK PROTEIN 70 (HSP70)* genes is induced by both heat and cold stress [37]. Yin *et al*. identified 93 *Homeodomain–Leucine Zipper* (*HD*-*Zip)* genes in *Brassica rapa*, 96 in *Brassica oleracea*, and 184 in *Brassica napus*. Among these genes, the *HD*-*Zip* gene *BraA09g011460.3C* was found to regulate carotenoid synthesis in rapeseed under high-temperature conditions [38]. Liu *et al*. investigated the dynamic characteristics of the DNA methylation system in NHCCs under HS [39]. However, prior research on Brassica HS responses overlooked both exposure duration and circadian effects.

In our study, high-resolution time-resolved transcriptome sequencing was conducted on NHCC leaves to analyze the gene expression dynamics in response to varying durations of HS and its interaction with the circadian rhythm. RNA sequencing (RNA-seq) analysis revealed a total of 13 863 differentially expressed genes (DEGs). DEGs on the first and fourth days of HS treatment were clustered, with each day’s DEGs being divided into 10 clusters. Enrichment analysis of these clusters revealed that the main physiological activities of NHCC on Day 1 at 43°C were the perception of external stimuli and signal transduction, whereas those on Day 4 at 43°C were primarily related to antioxidant metabolism and the onset of leaf senescence. Weighted gene coexpression network analysis (WGCNA) of genes meeting the criteria in all the samples revealed a correlation between circadian rhythm and plant responses to HS. Moreover, compared with wild-type plants, *BcCCA1*-overexpressing (*BcCCA1*-OX) plants presented greater heat susceptibility, indicating the role of *BcCCA1* as a suppressor of HS in NHCC. Moreover, consecutive studies have demonstrated the role of *BcCCA1* in modulating the heat resistance of NHCC through specific binding to the *BcHSFA2* promoter and suppression of its gene expression. This study demonstrated the differential responses of NHCC to varying durations of HS and confirmed that the core clock gene *BcCCA1* directly regulates the plant response to high temperature in NHCC. These results reveal new molecular pathways involved in plant HS adaptation.

## Results

### Transcriptome dynamics in NHCC under heat stress

**Figure 1:**
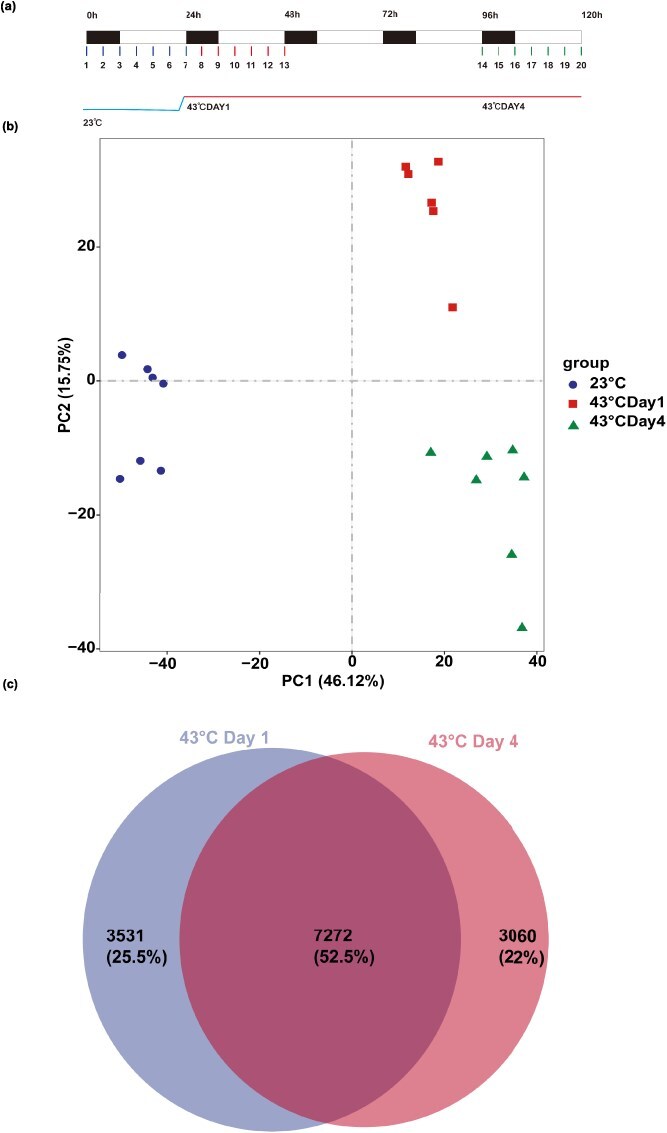
Clock response under high temperature in NHCC. (a) A total of 20 sampling time points are marked using circle dots of different colors. Sampling Points 1–7 indicate sampling every 4 h over a 24-h period at 23°C, after which the samples were immediately transferred to an incubator at 43°C. Sampling continued on Day 1 at 43°C and on Day 4 at 43°C. The black box indicates the 8 h dark, and the white box indicates the 16 h light. (b) PCA of the RNA-seq transcript data. PC1 and PC2 represent principal components 1 and 2, respectively. Three biological replicates per treatment were analyzed. (c) Number and percentage of DEGs that were significantly DE on both Days 1 and 4 at 43°C and unique to Days 1 and 4.

To explore how circadian rhythms influence HS responses, we conducted time-course RNA-seq on NHCC leaves during the four-leaf stage. The plants were initially cultivated at 23°C before being shifted to 43°C, allowing us to monitor changes in gene expression across the entire transcriptome over time. The leaf samples collected every 4 h on the final day at 23°C and on Days 1 and 4 at 43°C are shown in Fig. 1a. Following library preparation, RNA-seq was carried out on the Illumina NovaSeq platform using a paired-end 150-bp (PE150) configuration. Each sample yielded a minimum of ~14 million raw reads. When we analyzed gene expression patterns across all 20 time points using principal component analysis (PCA), the results clearly revealed that temperature (accounting for 46.12% of the variation) and duration (explaining 15.75% of the variation) emerged as the key drivers of transcriptional changes (Fig. 1b).

To assess the quality of the transcriptome data, we first checked the expression patterns of selected heat-responsive genes and circadian clock genes. Previous studies have demonstrated that HS significantly induces the expression of the Bcl-2-associated athanogene family gene *BAG6*, heat shock protein genes (*HSP*s), heat shock transcription factors (*HSF*s), and calcium-dependent protein kinases (*CDPK*s). Our RNA sequencing results validated the upregulation of *BcBAG6*_A05, *BcHSP70*_A01, *BcHSFA2*_A03, and *BcCDPK9*_A06 with increasing temperature (Fig. S1). The circadian clock genes *BcCCA1*_A05, *BcTOC1*_A09, *BcPRR7*_A10, and *BcPRR9*_A09 are homologous to the core *Arabidopsis* clock genes *CCA1*, *TOC1*, *PRR7*, and *PRR9*, respectively. These observed expression patterns are consistent with those of previous reports. Furthermore, the results of quantitative real-time polymerase chain reaction (qRT-PCR) confirmed the strong consistency between the expression patterns of these eight genes and the RNA-seq data. Thus, our RNA-seq data are suitable for subsequent analysis (Fig. S1).

We analyzed gene expression changes between different temperatures (i.e. 43°C versus 23°C) at the same Zeitgeber time points. Significant s were identified with a threshold |log_2_ (fold change) | ≥ 1 and a *P*-adjusted value <0.01. Consequently, we identified 13 863 genes with differential expressions in one or more comparison groups. To investigate the differences between short- and long-term thermal acclimation responses following high-temperature treatment, DEGs on Days 1 and 4 in the 43°C treatment were compared. The results revealed that 7272 genes (52.5%) were commonly differentially expressed, and the remaining DEGs were specific to either Day 1 (3531, 25.5%) or Day 4 (3060, 22%). Therefore, the changes in gene expression during the HS treatment exhibited three distinct temporal patterns: early (differentially expressed only on Day 1), persistent (differentially expressed on both Days 1 and 4), and late (differentially expressed only on Day 4). These distinct gene expression patterns suggest different roles in high-temperature perception, initial thermal response, and thermophysiological adaptation.

### Temporal clustering of gene expression and pathway enrichment analysis uncovers major metabolic and signaling adaptations to heat stress in NHCC

**Figure 2:**
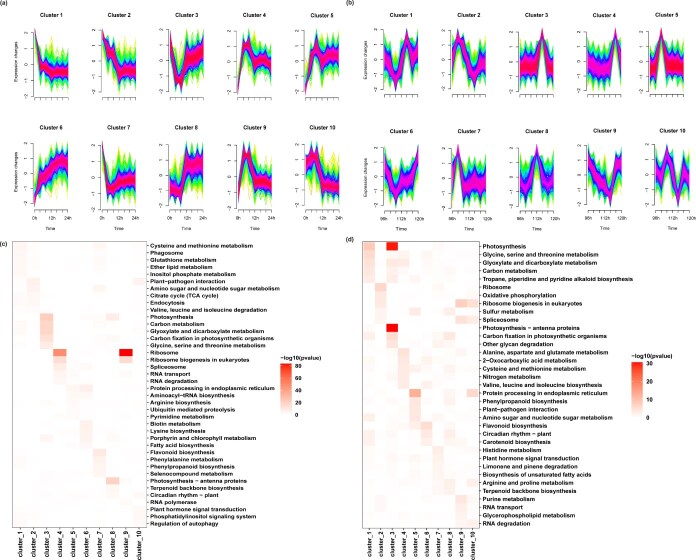
Mfuzz clustering DEGs at 43°C on Day 1 and 43°C on Day 4 on the basis of their expression patterns. (a) DEGs in the 43°C Day 1 cluster. (b) DEGs in the 43°C Day 4 cluster. The ordinate represents the fold changes in DEGs. The abscissa represents the sampling point. (c) KEGG pathway enrichment in each cluster at 43°C on Day 1 (*P*-adjusted <0.05). (d) KEGG pathway enrichment in each cluster at 43°C on Day 4 (*P*-adjusted <0.05).

**Figure 3:**
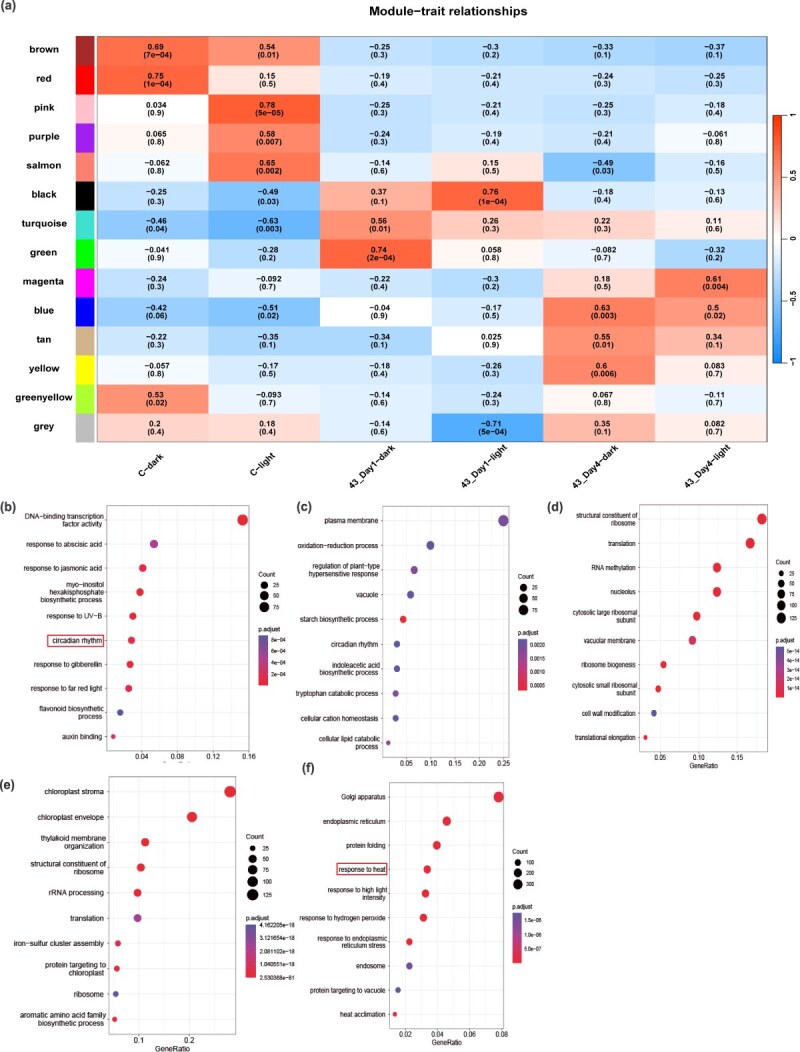
WGCNA and GO enrichment analysis. (a) Module–trait relationships. The numbers in each grid are the Pearson correlation coefficients and significant *P-*values in brackets. (b) GO enrichment analysis of the red module. (c) GO enrichment analysis of the pink module. (d) GO enrichment analysis of the green module. (e) GO enrichment analysis of the black module. (f) GO enrichment analysis of the blue module (Gene Ratio, the ratio of the number of genes enriched for each GO term to the total number of genes analyzed by GO. Pathways meeting the condition of *P*-adjust ≤0.01 were defined as significantly enriched pathways).

To analyze how gene expression patterns changed over time under HS, clustering analysis was performed using R-Pack-Mfuzz to identify sets of DEGs at different time points on Days 1 and 4 (Table S1). DEGs from both days were subsequently classified into 10 distinct clusters (Fig. 2a and b). Enriched Kyoto Encyclopedia of Genes and Genomes (KEGG) pathways within each cluster were identified (*P* < 0.05) and visualized as a heatmap (Fig. 2c and d). On Day 1 at 43°C, some pathways were specifically enriched. These included ‘phosphatidylinositol signaling system’ in Cluster 10, ‘citric acid cycle (TCA cycle)’ in Cluster 2, ‘glutathione metabolism’ in Cluster 1 and ‘porphyrin and chlorophyll metabolism’ in Cluster 6 (Fig. 2c). On Day 4 at 43°C, ‘oxidative phosphorylation’ in Cluster 2, ‘nitrogen metabolism’ in Cluster 4, and ‘carotenoid biosynthesis’ in Clusters 1 and 6 were enriched (Fig. 2d). Notably, the ‘ribosome’ pathway was the most significantly enriched on Day 1 at 43°C, whereas the ‘Photosynthesis-antenna proteins’ pathway was the most prominently enriched on Day 4 at 43°C (Fig. 3c and d).

### Integrative WGCNA reveals circadian and heat stress regulatory networks in the NHCC transcriptome

To explore the relationship between HS and circadian rhythms, this study analyzed an RNA sequencing dataset of 48 158 genes from 60 samples. To increase the steadiness of network construction, we initially filtered out genes showing zero expression across all the samples. Next, those genes with a median absolute deviation (MAD) in the top 75% and MAD values >0.01 (31 280 genes) were screened for subsequent WGCNA. Finally, we obtained 14 modules (Fig. 3a, Table S2).

Following the generation of characteristic gene maps for each module, coexpressed genes were analyzed using gene ontology (GO) enrichment analysis. This analysis focused on genes within modules that exhibited the most significant positive correlations with each subgroup. A bubble chart highlights the GO terms featuring only the 10 most significant entries with adjusted *P-*values <0.01. As depicted in Fig. 3a, the red module exhibited the strongest positive correlation (*r*^2^ ≥ 0.75) with the normal-temperature dark period. This module’s coexpressed genes showed a notable overrepresentation of GO terms linked to light-induced reactions and circadian regulation, specifically including the term ‘response to UV-B’ (Fig. 3b). The pink module exhibited the strongest positive correlation with the ambient light period (*r*^2^ ≥ 0.78), with its coexpressed genes significantly enriched in GO terms related to ‘starch biosynthetic process’, ‘cellular lipid catabolic process’, and ‘regulation of plant-type hypersensitive response’. Notably, the ‘circadian rhythm’ GO term was also significantly enriched in the pink module, suggesting that circadian clock genes are widely expressed during both the dark and light periods at 23°C (Fig. 3c). The green module exhibited the strongest positive correlation with the dark period on Day 1 of HS (*r*^2^ = 0.74), and coexpressed genes were notably enriched in GO terms linked to ‘translation’, ‘RNA methylation’, and ‘ribosome biogenesis’. These findings suggest that genes coexpressed within the green module may play a role in translation and RNA modification (Fig. 3d). The black module exhibited the strongest positive correlation with the light period on Day 1 of HS (*r*^2^ = 0.76), with coexpressed genes significantly enriched in GO terms such as ‘translation’ and ‘rRNA processing’(Fig. 3e). The blue module was significantly enriched for genes associated with GO terms such as ‘protein folding’, ‘response to heat,’, and ‘response to hydrogen peroxide’ (Fig. 3f). These results suggest that genes coexpressed within the blue module were primarily induced by HS. The magenta module showed the greatest positive correlation with the light period on Day 4 of the HS (*r*^2^ = 0.61). We did not analyze GO enrichment in the magenta module because the GO enrichment results of coexpressed genes in the magenta module revealed that the *P*-adjust was >0.1.

We found genes significantly enriched for the GO term ‘circadian rhythm’ in the red module (Fig. 3b) and ‘response to heat’ in the blue module (Fig. 3f). The results revealed that the red module contained the circadian clock core gene *BcCCA1*, the circadian rhythm regulatory gene *SUPPRESSOR OF PHYA-105 1(BcSPA1)*, and the light signal regulatory genes *BcRVE1*, *BcRVE6*, and *HY5 HOMOLOG* (*BcHYH)*. Thirteen *HSF*s factors were identified in the blue module (Table S3).

### BcCCA1 directly inhibits expression by interacting with its promoter

**Figure 4:**
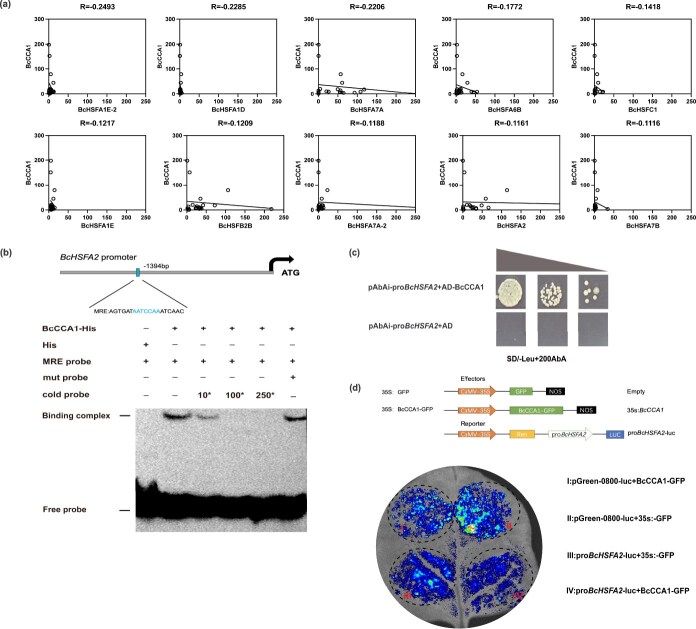
BcCCA1 interacts with the promoter of *BcHSFA2* and represses its transcription (a) Correlations between *BcCCA1* and HSFs (b) The EMSA results confirmed that BcCCA1 binds to the MRE motif in the promoter of *BcHSFA2*. (c) Y1H assays of BcCCA1 and the promoter of *BcHSFA2*. (d) Dual luciferase assays that BcCCA1 represses the transcription of *BcHSFA2*.

To investigate the regulatory role of circadian rhythms in the response of NHCCs to HS, we examined the relationship between the MYB transcription factor *BcCCA1* in the red module and HSFs in the blue module. Correlation analysis between the red and blue expression matrices revealed a negative correlation between *BcCCA1* and most HSFs (Fig. 4a). Subsequent analysis of the promoter elements of the *HSF*s in the blue module revealed an MYB-binding site (MRE) in the promoter region of *BcHSFA2* (Fig. 4b). These findings prompted further investigation into the interaction between BcCCA1 and the promoter of *BcHSFA2* (pro*BcHSFA2*).

To further validate the ability of BcCCA1 to directly bind to pro*BcHSFA2*, a yeast one-hybrid (Y1H) assay was performed. Y1H assays revealed that BcCCA1 interacted with pro*BcHSFA2* (Fig. 4c). Luciferase reporter assays indicated that BcCCA1 inhibited the promoter activity of *BcHSFA2* (Fig. 4d). Electrophoretic mobility shift assays (EMSAs) confirmed that BcCCA1 is directly bound to the MRE of pro*BcHSFA2* (Fig. 4b).

### *BcHSFA2* enhances NHCC thermotolerance

**Figure 5:**
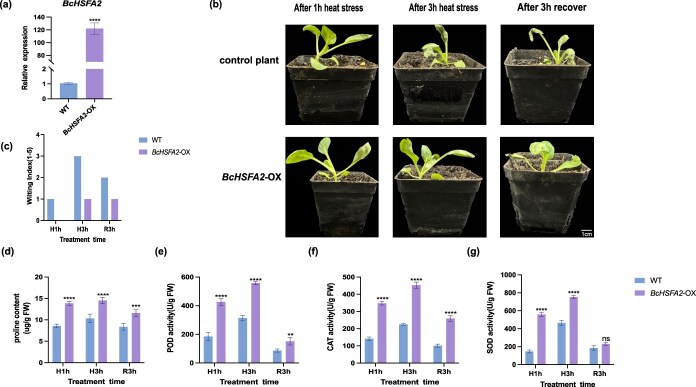
*BcHSFA2* improves heat tolerance in NHCCs. (a) Relative expressions of *BcHSFA2* in *BcHSFA2*-OX and control plants. (b) Phenotypes of *BcHSFA2*-OX and control plants before and after 3 h of heat treatment and 3 h of restoration to normal temperature. (c) WI of *BcHSFA2*-OX and control plants. (d) Proline contents in *BcHSFA2*-OX and control plants. (e) POD activities in *BcHSFA2*-OX and control plants. (f) CAT activities in *BcHSFA2*-OX and control plants. (g) SOD activities in *BcHSFA2*-OX and control plants (H1h and H3h represent treatment at 43°C for 1 and 3 h, respectively, and R3h represents recovery at 23°C for 3 h). Two-tailed Student’s *t-*test, *P* < 0.01. Scale bar, 1 cm.

To investigate the role of *BcHSFA2* in NHCC, an overexpression vector was constructed and transiently expressed in NHCC leaves. The overexpression of *BcHSFA2* was confirmed by qRT–PCR analysis (Fig. 5a). Subsequently, the *BcHSFA2*-OX plants were subjected to high-temperature treatment (43°C), and their phenotypes were monitored after 1 and 3 h, after which they were restored to room temperature (23°C). The results indicated that the *BcHSFA2*-OX plants displayed only slight wilting under high-temperature treatment, whereas the control plants exhibited more pronounced wilting. This observation was further supported by the quantitative wilting index (WI) analysis. The WI is a 0–5 scoring system used to evaluate the extent of heat-induced leaf wilting, with higher scores indicating more severe damage. Consistently, *BcHSFA2*-OX plants showed lower WI values than wild-type (WT) plants across all treatment stages (H1h, H3h, and R3h), reinforcing the conclusion that *BcHSFA2* enhances thermotolerance (Fig. 5b and c). In pursuit of a deeper analysis into the influence of *BcHSFA2* on NHCC thermal endurance, the proline contents and the activities of CAT, SOD, and POD enzymes were measured in both *BcHSFA2*-OX plants and control plants. Compared with the controls, *BcHSFA2*-OX plants accumulated higher proline levels and showed increased activities of CAT, SOD, and POD (Fig. 5d–g).

### BcCCA1 negatively regulates NHCC thermotolerance

**Figure 6:**
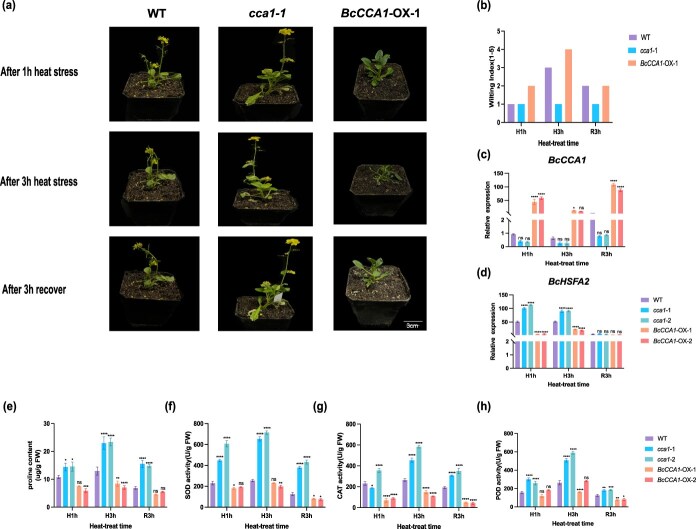
BcCCA1 negatively regulates NHCCs response to high-temperature stress. (a) Phenotypes of the WT, *cca1*, and *BcCCA1*-OX before and after 3 h of heat treatment and 3 h of restoration to normal temperature. (b) WI of the WT, *cca1*, and *BcCCA1*-OX (c) Relative expressions of *BcCCA1* in *BcCCA1*-OX and *cca1.* (d) Relative expressions of *BcHSFA2* in *BcCCA1*-OX and *cca1*. (e) Proline contents in the WT, *BcCCA1*-OX, and *cca1*. (f) SOD activities in the WT, *BcCCA1*-OX, and *cca1*. (g) CAT activities in the WT, *BcCCA1*-OX, and *cca1*. (h) POD activities in the WT, *BcCCA1*-OX, and *cca1* (H1h and H3h represent treatment at 43°C for 1 and 3 h, respectively, and R3h represents recovery at 23°C for 3 h). Two-tailed Student’s *t*-test, *P* < 0.01. Scale bar, 3 cm.

To confirm the involvement of *BcCCA1* in the reaction of NHCC to HS, we generated stable transgenic plants overexpressing *BcCCA1* (*BcCCA1*-OX) (Fig. S3) and used CRISPR/Cas9 technology to knock out *BcCCA1* in NHCC (*cca1, Sequencing* of the transgenic plants revealed that both the ‘*cca1-*1’ and ‘*cca1-*2’ mutants harbored mutations in the first exon. Specifically, ‘*cca1-*1’ contained a large-fragment deletion, while ‘*cca1-*2’ contained a 1-bp deletion coupled with a single-nucleotide substitution.) (Fig. S4). Two independent *BcCCA1*-OX lines and two independent cca1 mutant lines were selected for analysis. These plants were subjected to HS for 1 h (HS1h) and 3 h (HS3h), followed by recovery at normal temperature for 3 h (R3h). Under these conditions, the cca1 mutants consistently displayed more heat-tolerant phenotypic traits, which was further supported by their uniformly low WI (WI = 1, 1, and 1 at HS1h, HS3h, and R3h). In contrast, *BcCCA1*-OX plants not only exhibited more severe heat-induced wilting, as reflected by their higher WI index (2, 4, and 2), but also developed stunted growth with a thin, short stature (Fig. 6a and b). We compared the expression levels of *BcCCA1* and *BcHSFA2* in *BcCCA1*-OX plants, cca1 mutants, and the WT. At HS1h, the expression level of *BcCCA1* in *cca1* mutants was significantly lower than in the WT, whereas its expression in *BcCCA1*-OX plants was markedly higher (Fig. 6c). Notably, at HS3h, its expression in *cca1-*1, *cca1-*2, and *BcCCA1*-OX-2 did not differ significantly from that in the WT. Although its expression in *BcCCA1*-OX-1 was slightly higher than in the WT, all values were lower than those observed at HS1h (Fig. 6c). After 3 h of recovery (R3h), the expression level of *BcCCA1* expression increased in all plants; however, its expression in cca1 mutants remained significantly lower than in the WT, whereas its expression in *BcCCA1*-OX plants was significantly higher. Moreover, the expression of *BcHSFA2* was strongly suppressed in the *BcCCA1*-OX line following heat treatment (Fig. 6d). In addition, we detected the proline contents and the activities of CAT, SOD and POD in the WT, *BcCCA1*-OX and *cca1* mutants at different time points (Fig. 6e–h). Compared with WT, *cca1* mutants showed higher proline levels and greater antioxidant enzymes activities, whereas both parameters were reduced in the *BcCCA1*-OX plants. These results demonstrate that overexpression of *BcCCA1* reduced heat resistance, whereas the heat resistance of the *cca1* mutant was significantly better than that of the WT.

## Discussion

High-temperature stress is an increasingly severe environmental constraint for plants [40]. In Arabidopsis, links between circadian regulation and thermotolerance are well established, with core clock components such as *EARLY FLOWERING 3* (*ELF3)*, *PRR7*, and *PRR9* contributing to temperature compensation and transcriptional reprogramming [41, 42]. Our study extends these insights to NHCCs, revealing dynamic transcriptional responses and identifying a regulatory module that integrates circadian control with heat stress adaptation.

Time-series transcriptome sequencing revealed 13 863 DEGs under HS, of which 3531 genes were specific to Day 1 and 3060 genes were specific to Day 4. (Fig. 2c, Table S1). Comparisons with earlier datasets under heat and darkness revealed 6575 DEGs with no significant variation [43], but an additional 5812 novel DEGs emerged, emphasizing that both the duration of HS exposure and circadian rhythm shape transcriptional outputs. Temporal clustering and KEGG enrichment further demonstrated stage-specific responses. On Day 1, the ‘phosphatidylinositol signaling system’ was enriched (Fig. 2c), mediating Ca^2+^ influx and triggering phosphorylation cascades [44, 45]. Consistent with this pattern, *PHOSPHOLIPASE C*s (*BcPLC*s), *MULTIPROTEIN BRIDGING FACTOR 1C* (BcMBF1C), and the calmodulins, including *CALMODULIN-LIKE PROTEIN 4* (*BcCML4*) and *CALMODULIN 3* s (*BcCAM3*s), were strongly induced, indicating rapid activation of calcium-mediated signaling pathways [46–49]. In parallel, the ‘glutathione metabolism’ pathway was upregulated (Fig. 2c), with glutathione transferases rapidly induced and glutathione peroxidases maintaining high expression, underscoring the central role of reactive oxygen species (ROS) detoxification in the initial phase [50]. The enrichment of autophagy-related pathways during this stage further supports their protective role in thermotolerance [51]. These findings suggest that early responses to HS rely on the coordinated activation of signaling, detoxification, and recycling mechanisms.

By Day 4, enrichment patterns shifted toward prolonged metabolic adaptation. ‘Oxidative phosphorylation’ was strongly enriched (Fig. 2d), reflecting increased ROS accumulation during extended HS [52]. The expression of ROS scavengers, including *SUPEROXIDE DISMUTASE*s (*BcSOD*s), *GLUTATHIONE PEROXIDASE*s (BcGXPs), *PEROXIDASE*s (*BcPOD*s), and *DEHYDROASCORBATE REDUCTASE* (*BcDHAR*), peaked at this stage, whereas other antioxidant enzyme genes, such as *ASCORBATE PEROXIDASE*s (*BcAPX*s), *ASCORBATE OXIDASE B* (*BcAOXB*), and *SUPEROXIDE DISMUTASE 2* (*BcSOD2*), exhibited transient early induction before returning to baseline expression levels (Fig. S5, Table S4). This finding indicates a biphasic detoxification strategy in which some genes buffer acute ROS surges, whereas others sustain long-term oxidative homeostasis. Additional enrichment of ‘carotenoid biosynthesis’, ‘purine metabolism’, and ‘arginine and proline metabolism’ further highlights antioxidant reinforcement under prolonged HS. ‘Nitrogen metabolism’ was also induced, with significant upregulation of *GLUTAMINE SYNTHETASE 1* (*GS1*) and *ASPARAGINE SYNTHETASE 1* (*AS1*), which is consistent with enhanced nitrogen remobilization and leaf senescence [53]. Photosynthesis-related pathways were impaired on Day 4, yet protective upregulation of antioxidant enzymes (SOD, APX, and POD) and HSP21 family proteins was observed, supporting photosynthetic stability under stress [54] (Table S5).

Hormone signaling contributed an additional regulatory layer. Genes related to the abscisic acid (ABA), brassinosteroid (BR), ethylene, and jasmonic acid (JA) pathways were differentially expressed (Fig. S5, Table S4). Early induction of *PROTEIN PHOSPHATASE 2C* (*BcPLY7*), *BRASSINOSTEROID-6-OXIDASE 2* (*BcBR6OX2*), *1-AMINOCYCLOPROPANE-1-CARBOXYLATE OXIDASE 5* (*BcACO5*), *ETHYLENE RESPONSE FACTOR 1* (*BcERF1*), and *LIPOXYGENASE 5* (*BcLOX5*) reflected broad hormonal activation during initial HS [55–64]. Moreover, the downregulation of the negative regulators *BRASSINOSTEROID-INSENSITIVE 2* (*BcBIN2*) in the BR pathway and *JASMONATE ZIM-DOMAIN PROTEIN 3* (*BcJAZ3*) in the JA pathway facilitated the transcription of stress-related factors [65, 66]. These results indicate that hormonal crosstalk rapidly reconfigures signaling networks to fine-tune thermotolerance.

Hormone signaling contributed to an additional regulatory layer. Genes related to the ABA, BR, ethylene, and JA pathways were differentially expressed (Fig. S5, Table S4). Early induction of *PP2C* (*BcPLY7*), *BcBR6OX2, BcACO5*, *BcERF1*, and *BcLOX5* reflected broad hormonal activation during initial HS [55–64]. Moreover, the downregulation of the negative regulators *BcBIN2* (BR pathway) and *BcJAZ3* (JA pathway) facilitated the transcription of stress-related factors [65, 66]. These results indicate that hormonal crosstalk rapidly reconfigures signaling networks to fine-tune thermotolerance.

Circadian regulation was also broadly perturbed. Nearly all circadian genes, including *BcTOC1*, *BcPRR7*, and *BcPRR9*, were altered under HS. (Fig. S5, Table S4). Such changes suggest reprogramming of the circadian system to accommodate prolonged stress. Among these, we identified the BcCCA1–BcHSFA2 axis as a critical regulatory module. These functions are consistent with the established role of *HSFA2* in stabilizing proteins through HSPs, whereas the accumulation of proline represents a typical physiological response of plants under stress, serving not only as a direct scavenger of ROS but also as an indirect protector against oxidative damage by modulating the cellular redox state (Fig. 5) [67, 68]. Importantly, BcCCA1 was shown to negatively regulate thermotolerance in nonheading Chinese cabbage by directly binding to the MRE motif in the *BcHSFA2* promoter and repressing its transcriptional activity (Figs 4 and 6). This interaction positions *BcCCA1* as a circadian gatekeeper of heat response initiation.

The role of *HSFA2* in thermomemory further strengthens this regulatory link. Previous studies have demonstrated that *HSFA3* is targeted by CCA1, with increased expression in *cca1*/*lhy* mutants [69, 70], and that the expression of *HSFA2* is maintained for several days following HS [71]. Our data confirmed sustained *BcHSFA2* expression during recovery (Fig. S1a), which was likely mediated by autoregulation, post-translational modifications [72, 73], and chromatin-based memory [72]. While the acute HS assays in this study revealed short-term regulatory events, transcriptome profiling over 4 days highlights their potential impact on long-term acclimation. The enhanced thermotolerance observed in *cca1* mutants likely results from stronger initiation of protective transcriptional networks, which could provide survival advantages under chronic HS.

While our functional analyzes utilized an acute HS (43°C for 1–3 h) to assess phenotypic differences, our transcriptome data encompassed a prolonged stress duration (4 days). The identification of the BcCCA1-BcHSFA2 module, which governs the initial heat shock response, provides insight into the early regulatory events that may influence long-term adaptation. Given the established role of *HSFA2* in thermal tolerance, the enhanced acute thermotolerance observed in the cca1 mutant likely stems from a more robust initiation of the protective transcriptome, which could contribute to enduring prolonged stress. Future investigations employing repeated heat priming or continuous moderate heat stress will be valuable to directly elucidate the role of this module in long-term acclimation.

**Figure 7:**
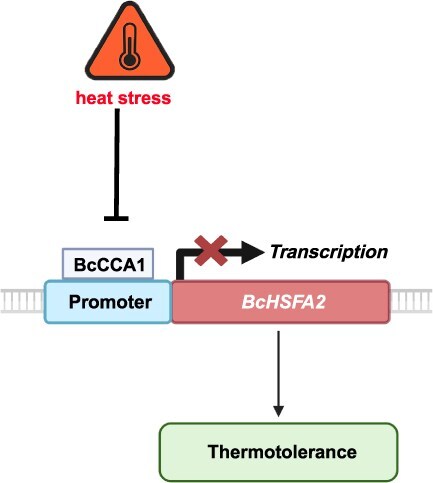
Schematic representation of the regulatory relationship between BcCCA1 and *BcHSFA2* in NHCC under HS. HS suppresses *BcCCA1* expression, which relieves its inhibitory effect on *BcHSFA2*, leading to elevated *BcHSFA2* transcription and enhanced heat tolerance. Arrowheads and T-bars denote activation and repression, respectively

Taken together, these findings reveal a stage-specific transcriptional strategy in NHCCs: early responses dominated by calcium signaling, antioxidant metabolism, and autophagy and late responses characterized by ROS detoxification, redox homeostasis, nitrogen remobilization, and photosynthetic adjustment. Most importantly, we identified the BcCCA1–BcHSFA2 module as a novel regulatory axis that links circadian rhythm with thermotolerance. This discovery not only advances our understanding of circadian–stress crosstalk but also provides a mechanistic framework for plant thermomemory and long-term acclimation. By directly repressing *BcHSFA2*, BcCCA1 modulates the onset of protective responses, thereby shaping both acute and sustained adaptation (Fig. 7). These insights suggest that the manipulation of circadian–heat stress interactions could represent a promising strategy for engineering crop thermotolerance under future climate conditions.

## Materials and methods

### Plant material and growth conditions

Seeds of ‘Suzhouqing’ and ‘49-CX’ were provided by the Laboratory of System Biology of Cabbage, Nanjing Agricultural University (Nanjing, China). The seeds were sown in seedling trays filled with a 3:1 mixture of matrix and vermiculite and placed in a controlled growth chamber. The growth conditions were maintained at 23°C with a 16 h light/8 h dark photoperiod and 55% relative humidity.

### Sample collection and heat stress treatment

Seedlings at the four-leaf stage were used for sampling. Samples were collected every 4 h during the final 24 h at 23°C and on Days 1 and 4 after transferring to 43°C, yielding a total of 21 time points (Fig. 1a). Day 1 and 4 sampling periods represented the transcriptomic features of the acute shock phase and long-term adaptation phase of HS, respectively. For each time point, three independent biological replicates were obtained, resulting in a total of 63 samples. All replicates were maintained in the same growth chamber to minimize variability in light intensity and spectrum, and trays were randomly arranged to reduce potential microclimatic effects. During functional validation of the *BcCCA1* gene, all heat treatments were initiated at the beginning of the light period to control for circadian influences, ensuring that wt, mutant, and overexpressing plants were exposed to HS at the same circadian phase. The leaf samples were rapidly frozen in liquid nitrogen and stored at −80°C for RNA and cDNA preparation.

### RNA sequencing

Total mRNA from NHCCs was extracted using TRIzol reagent, and its concentration and purity were assessed with a NanoDrop 2000 (Thermo Scientific). RNA integrity was evaluated using an Agilent 2100 Bioanalyzer, and all the samples had RIN values >7. Qualified mRNA samples were subjected to next-generation sequencing (NGS) on the Illumina NovaSeq platform (Shanghai Personal Biotechnology Co., Ltd, China) with 150-bp paired-end reads. For each sample, 36–52 million reads were obtained, with the transcriptome coverage ranging from 94.05% to 95.07%. Image files generated by the sequencer were processed using the platform’s software and converted into FASTQ format. Raw reads were filtered with Cutadapt (v1.15) to remove adapters and low-quality sequences, resulting in the generation of high-quality clean data. The reference genome and gene annotation files were obtained from the Laboratory of System Biology of Cabbage, Nanjing Agricultural University.

### Sequencing data analysis and identification of differentially expressed genes

Gene expression was quantified as raw read counts using HTSeq (v0.9.1) and normalized as FPKM. Differential expression analysis was performed using DESeq2 with thresholds of |log2FoldChange| > 1 and adjusted *P-*value <0.01. Principal component analysis was conducted with Genes Cloud Tools, and clustering analysis was performed using the Mfuzz package in R. KEGG enrichment analysis was based on the Mfuzz clustering results, and the most enriched pathways were visualized using R. WGCNA was performed with 60 samples, focusing on 31 280 genes in the top 75% median deviation and MAD >0.01. The scale-free topology criterion was met with a soft-thresholding power of β = 26 (Fig. S2a). Gene clustering trees were generated from expression correlations, and modules were assigned unique colors, with unclustered genes shown in gray (Fig. S2b). Module gene distributions are presented in Fig. S2c. GO enrichment bubble plots were generated with the clusterProfiler R package.

### Quantitative real-time PCR validation

Nine randomly selected DEGs from the RNA-seq data were validated by qRT–PCR using Hieff^®^ qPCR SYBR Green Master Mix (Yeasen, Shanghai, China). BcGAPC (BraC08g031360) was used as the reference gene. Relative expression levels were calculated using the 2^−ΔΔCt^ method. The primers used are listed in Table S6.

### Vector construction and transgenic plant generation

A BcCCA1-GFP overexpression vector was constructed and introduced into Agrobacterium. Transformation into ‘49-CX’ via Agrobacterium-mediated transfer resulted in the generation of BcCCA1-overexpressing plants [74]. The primer sequences are provided in Table S6.

### CRISPR/Cas9-mediated BcCCA1 knockout mutant generation

A single guide RNA (sgRNA: GGACTGAGGAAGAACATAAT AGG) was designed using CRISPOR (http://crispor.tefor.net). Complementary oligonucleotides were annealed into double-stranded sgRNA and ligated into the BbsI-digested pMD18-T vector. After sequencing verification with the primer pAC008-R, the fragment was cloned and inserted into pCAMBIA1301 following Hind III and Kpn I digestion. The construct was subsequently transformed into Agrobacterium strain GV3101 to generate the *BcCCA1* knockout mutant (*cca1*) [74]. Genomic DNA was extracted from resistant plants, and the target region was amplified and sequenced (Sangon Biotech, Nanjing, China). Mutations were analyzed using SnapGene. The primer information is provided in Table S6.

### Yeast one-hybrid assay

The promoter of *BcHSFA2* was cloned and inserted into the pAbAi vector, whereas *BcCCA1* was cloned and inserted into pGADT7. The recombinant plasmids were subsequently cotransformed into Y1H Gold yeast cells, after which the transformants were screened on SD/-Ura/-Leu media supplemented with AbA. The primers used are listed in Table S6.

### Dual-luciferase assay

The coding sequence (CDS) of *BcCCA1* was cloned and inserted into pR101-GFP, and the promoter of *BcHSFA2* was cloned and inserted into pGreen-0800. The constructs were subsequently transformed into *Agrobacterium tumefaciens* GV3101 containing pSoup. A bacterial suspension mixture was infiltrated into tobacco leaves, which were incubated for 60–72 h in the dark under ambient conditions. Luciferase activity was measured using the Dual-Luciferase Reporter Assay System (Yeasen, Shanghai, China). The primers used are listed in Table S6.

### Electrophoretic mobility shift assay

The CDS of *BcCCA1* was cloned and inserted into the pCold-His vector, and the recombinant protein was purified using Ni-agarose His-Tag Protein (Yeasen, China). Biotin-labeled oligonucleotide probes were synthesized (Tsingke, China) and annealed into double-stranded DNA (95°C for 3 min, cooled at 1°C/90 s to 25°C, stored at −20°C). EMSAs were performed with a chemiluminescence detection kit (Beyotime, China). Briefly, 0.1 μmol of labeled probes was incubated with or without protein for 20 min at room temperature, and the complexes were resolved by PAGE and transferred to nylon membranes. Detection was performed with the manufacturer’s reagents using a ChemiDoc MP imaging system (Bio-Rad, USA). Probe sequences are listed in Table S6.

### Wilting index quantification

Heat-induced wilting was quantified using a five-point WI following previously described methods with minor modifications. WI scores were assigned based on the extent of leaf drooping as follows: 0 = no visible wilting; 1 = <25% of leaves slightly drooping; 2 = 25%–50% of leaves visibly drooping; 3 = 50%–75% drooping with leaf softening; 4 = >75% leaves collapsed; 5 = plant nearly dead. For each genotype and treatment (H1h, H3h, and R3h), representative plants were photographed and scored. Mean WI values are presented in Figs 5 and 6, and WI scoring criteria are provided in Table S7.

### Determination of SOD, POD, and CAT activity

The activities of SOD, POD, and CAT in WT, *BcCCA1*-OX, and mutant plants before and after HS treatment were measured using commercial kits (Solarbio, China). The results are expressed as U/g fresh leaf tissue.

## Supplementary Material

Web_Material_uhag033

## Data Availability

The data that support the findings of this study will be available in NCBI at (https://dataview.ncbi.nlm.nih.gov/object/PRJNA1282857?reviewer=toselqsndcsesnqickjshkfh36).
